# The Germ Theory of Dental Caries

**Published:** 1883-02

**Authors:** C. T. Stockwell

**Affiliations:** Conn.


ARTICLE III.

### THE GERM THEORY OF DENTAL CARIES.

BY DR. C. T. STOCKWELL, CONN.

Read before the Brooklyn Dental Society, January 8, 1883.

REVISED FOR THE INDEPENDENT PRACTITIONER.

I have thought that, perhaps, I could in no better way repay you for your cordial and greatly appreciated invitation to be present on this occasion, than by briefly considering some of the objections that have been raised against the germ theory as applied to the etiology of dental caries; or, perhaps I should say, some of the questions that have been propounded by those who have assumed the negative position since the first reading of the paper which was deemed worthy your attention and discussion at your last meeting.

And first, allow me to say that whatever our scientific men may finally demonstrate as the cause of dental caries, Prof. Mayr has, in my humble opinion, made it forever impossible to consider the action of acids upon the teeth as anything more than accidental. It is not the first step in true caries any more than a congenital defect, simple fracture, imperfect development, or any other favorable predisposing condition. The action of acids should be classed with these, and with these alone. Until something else takes place, caries proper cannot be said to have begun. Acids can and do result in the wasting away or gradual destruction of teeth, in rare cases, by the process of erosion, but that is a very different thing from caries. In fact, it has nothing in common with it in either its incipient or advanced stages. Caries does not begin until some portion of the organic structure of the tooth is attacked, and then its progress is commensurate with the destruction of the organic tissue. Disease of a tooth is disease of the organic, vitalized tissue, and that alone. In-organic material diseased? Think of it for a moment. What is the nature of the disorder common to lime-salts? What is its name? Tissue composed largely of lime-salts may, it is true, become diseased, but it is that portion called organic, be it large or small, that is subject to that condition. Thus we claim that the destruction of the organic precedes the breaking down of the lime-salts in dental caries. Read carefully the report of Prof. Mayr's analysis of decayed tooth substance, in the January number of the *New England Journal*, and see if the decay can be accounted for upon the basis of chemical action.

That the destruction of the organic part of the teeth precedes the breaking down of their lime-salts is, I think, clearly established by recent investigation. Admitting this statement to be true, the question at once arises as to the nature and character of the active agent. I know of but two hypotheses that have been set forth thus far—the inflammation and the germ theories. You are all, doubtless, much more familiar with the doctrine of that eminent school of gentlemen across the river than I am, but it seems to me to be hardly less satisfactory than the old acid theory. It is, indeed, a single step away from it, but nevertheless they seem to be holding on to their old acid friend with one hand, while they reach out the other, endeavoring to find something else to yoke up with it, to account for that phenomenon termed dental caries. For the discoveries of these gentlemen regarding the structural organization of the teeth, they deserve the gratitude of the entire dental world; but I must regard the failure of the inflammation theory to account for the decay of dead teeth and ivory teeth inserted in artificial dentures, as fatal to that system. In fact, one of these gentlemen, not long since, when asked to account for this fact, finally admitted that in dead teeth germs very likely were the active agents.

And this brings me, after so long a preamble, to some of the objections raised to the germ theory.

The first question is suggested by the foregoing sentence. "How can the germ theory be applied to dead teeth any more satisfactorily than the inflammation theory? Is not the organic matter already dead?" True, as regards a large mass of the fibrils, or the reticular bioplasson structure; but there remains the devitalized protoplasmic substance of the fibrils, and the connective tissue that holds in position the granules of lime-salt, all of which form legitimate pabulum for bacterial organisms. These, being chiefly deprived of their vital resisting power, fall an easier prey to the attacking enemy.

This harmonizes with the observation that dead teeth decay more rapidly than live teeth, especially when the outside enemy is not excluded by a filling that hermetically seals the cavity.

"But," it is said, "how strange it is that these organisms should chew up teeth when they have so much in the mouth besides to live on!"

In answer, let me say that it may seem more strange that coral should be "chewed up" by a similar organism, when the proportion of organic matter in coral is only about one-tenth of one per cent.

In Germany a cement is made and used for various purposes, in which milk curd is an ingredient, the proportion being about ninety per cent. of lime to ten per cent. of this organic curd. The only trouble in its practical use is said to be the fact that worms, veritable, visible worms, destroy it by eating into it. Its structure being destroyed, it is reduced to the granular state and falls to pieces. If visible worms destroy a substance whose organic proportion is only ten per cent., who shall say that microscopical organisms may not successfully attack a structure as dense even as enamel, much more exposed dentine? And right here let me say that the books are, very possibly, in error regarding the proportions of organic and in-organic matter of the teeth. Not long since it occurred to me that the analysis of tooth substance, as given us by the books, is made with the teeth in a dry state. To determine the accuracy of these statements, I suggested to Prof. Mayr that some experiments be made to ascertain the proportions of water in tooth substance. Some work has been done in that direction, but as it is not yet completed I can only give you the approximate results.

Take an average human tooth, in a normal condition, directly from the mouth, and the analysis of dentine, taking the water into account, would show about the following proportions: Lime-salts, forty per cent.; water, forty per cent., and organic material, twenty per cent.

While this statement can be relied upon as only approximately accurate, it must be admitted that, in our consideration of the agents at work in the accomplishment of the destruction of teeth, the natural amount of water, in the constitution of these organs, must be taken into account.

Water, taken alone, can hardly be said to be organic; but when it forms a constituent part of tissue, it is, in a material sense, organic.

If the relative proportions of lime-salts to the organic are, say, forty per cent. of lime-salt to sixty per cent. of organic material, instead of seventy-two per cent. of lime-salt to twenty-eight per cent. of organic, the teeth may be more easily "chewed up" than may have been supposed.

But this view of the case is by no means necessary to the disposal of the objection. The real answer may be stated somewhat as follows: Bacterial organisms, it is true, are present in all parts of the mouth, but they successfully attack the teeth because they are the weakest in resisting force. The organic portion of tooth structure is circumscribed. The circulation is, compared with the mucous membrane, much less and much slower, necessarily. In this fact lies the cause of a lesser vitality. The vascular tissues possess a greater resisting power because of their vascularity. By means and because of this greater circulation, the mucous membrane is enabled to overcome and even to absorb these attacking organisms. Arrest the circulation, and putrefactive processes are at once set up. Systemic disease often first expresses itself in a disturbed condition of the teeth. In speaking of this matter not long since, a medical gentleman remarked that "his teeth were his health thermometers," as they first indicated any slight constitutional disturbance.

Another question is the following: "If the germ theory is correct, why do not teeth of skeletons decay and crumble to atoms sooner?"

I might, with far more reason, ask why it is that the fibrils of the teeth are, alone, so disastrously affected by acids. The use of acid fruits, food substances and fluids has, to my knowledge, never been known to produce such direful results upon the mucous membrane of the mouth and stomach as, it is claimed, is the result when such acids come in contact with the fibrils of the teeth. Extracted teeth and the teeth of skeletons do not decay, because, first, the fibrils and connective tissue become so dessicated as to render them unsuitable for the normal development of germs; secondly, the temperature is below the standard necessary for their development.

Dead teeth in the mouth do decay rapidly, but there they have the important favoring conditions of temperature and moisture.

The question is again asked, "why is it that any stray germs left in the structure of the teeth beyond the line of excavation, and consequently sealed up in the tooth by the filling, do not multiply indefinitely and bring about an early renewal of decay?"

Our friend, Dr. Clark, can best answer this question. But that there is danger of this very thing occurring in cases where no antiseptic agent is used, or where the filling itself is not antiseptic, I am quite confident.

It may be said, however, that the life of these organisms is relatively very short, and that in cases where the supply from without is cut off by a filling that hermetically seals the cavity, the enclosed organisms soon die or are overcome, and in many cases even are absorbed by the interior vital forces which reside in the protoplasmic fibrils.

Very possibly, also, the matter of a non-supply of oxygen might come in here as a potent question to be considered, were we to attempt to discuss principles and processes. This question is one of no little practical importance and needs to be studied most carefully.

Our good friend, Dr. Atkinson, possibly builded better than he knew, when, some years since, he remarked that we should invariably bathe a cavity with carbolic acid just prevlous to filling, for "carbolic acid seems to be a friend to the teeth." Certainly, for the purpose indicated, few will deny that carbolic acid is "a friend to the teeth;" but just how carbolic acid performs this friendly act I have always been at a loss to explain, until the view presented itself to my mind that germs were an active agent in dental caries.

This whole subject opens out a wonderful field for observation, discussion, and patient, careful, scientific research. I have spent months, I might say years, in the endeavor to learn something of the great questions involved in this field of investigation, but it is no assumption of feigned modesty when the confession is freely made that the threshold is scarcely passed. I can only assert a willingness to learn, and, being no scientist myself, I can only rely upon such investigations of others as are open to all who choose to avail themselves of them.

Just before I left home, the *Ohio State Journal*, containing Dr. Watts' labored article in defence of the acid theory of caries, came to hand. I shall attempt no reply here. In fact, none is needed. I will only say that if, in an article of nineteen and one-half pages, confessedly the longest article he ever wrote, nothing better can be brought forward by its author in defence of his pet theory than sallies of witticisms, attempted ridicule, and the most unfair misconstruction of isolated sentences, then the germ theorists have nothing to fear from that quarter. His sole conclusion is a Scotch verdict—not proven. Not proven! when Drs. Underwood and Milles uniformly find bacteria present, and when Dr. W. D. Miller, himself not an advocate of the germ theory, says that he has nearly one thousand specimens, in every one of which they are present—specimens which have been examined by the very best authorities in Germany, who unanimously pronounce them bacteria? In the name of conscience, then, what testimony is conclusive?

In closing, I wish to reiterate two simple points that appear as the result of recent investigation. First, that careful chemical experiments show, most conclusively, that the histological structure of a tooth is destroyed before there is any loss of the inorganic material. That, at the border line, where the real fight is being carried on, the lime-salts are present in normal proportions; consequently, there can have been no solution of lime-salts. It shows that the structure is broken down, as the first step in caries. Secondly, microscopical investigations, those which I believe to be most reliable, and which, to my knowledge, remain unchallenged, show the uniform presence of micro-orgcnisms in the disintegrated masses at the border line of decayed and healthy tissue, and often, if not always, that they are found to a greater or less extent occupying the dentinal tubuli in advance of the breaking down of the structure at the border line. Numerous sections show them present in the tubuli at a depth of from one to two millimeters beyond what, to the eye, would appear to be the healthy line.

This much, it would seem, these investigations fully establish. But even granting all this, I am aware that many will contend that organisms are not proven to be the agent that destroys the organic structure.

Right here we leave what seems to me to be the absolute, and, as far as present investigations are concerned, have to content ourselves with the relative. So far as I am personally concerned, just how, or just why, a careful weighing of the relative has resulted in a conviction that germs are the agents that cause the destruction of the organic tissue, I will not weary you by attempting to describe. It is a matter that each one must investigate for himself. If anything that I have said should prove a further incentive to that original investigation which was so ably urged before you, not long since, by your secretary, Dr. Johnston, I shall have gained my full object.

